# Comparative transcriptome analysis of venom glands from *Cotesia vestalis* and *Diadromus collaris*, two endoparasitoids of the host *Plutella xylostella*

**DOI:** 10.1038/s41598-017-01383-2

**Published:** 2017-05-02

**Authors:** Wei Zhao, Min Shi, Xi-qian Ye, Fei Li, Xiao-wei Wang, Xue-xin Chen

**Affiliations:** 1 Institute of Insect Sciences, Zhejiang University, 866 Yuhangtang Road, Hangzhou, 310058 China; 2 Ministry of Agriculture Key Lab of Molecular Biology of Crop Pathogens and Insect Pests, Zhejiang University, 866 Yuhangtang Road, Hangzhou, 310058 China; 3 State Key Lab of Rice Biology, Zhejiang University, 866 Yuhangtang Road, Hangzhou, 310058 China

## Abstract

Venoms secreted by the venom gland (VG) of parasitoid wasp help ensure successful parasitism by host immune suppression and developmental regulation. *Cotesia vestalis*, a larval endoparasitoid, and *Diadromus collaris*, a pupal endoparasitoid, parasitize the diamondback moth (DBM), *Plutella xylostella*. To explore and compare the venom components of two endoparasitoids, we sequenced transcriptomes of the VGs and wasp bodies without VGs (BWVGs) of the two endoparasitoids. Statistically enriched GO terms and KEGG pathways of the two VGs compared to respective whole-body background were similar and reflected active protein biosynthesis activities in the two VGs. 1,595 VG specific genes of the *D*. *collaris* VG and 1,461 VG specific genes of the *C*. *vestalis* VG were identified by comparative transcript profiling. A total of 444 and 513 genes encoding potential secretory proteins were identified and defined as putative venom genes in *D*. *collaris* VG and *C*. *vestalis* VG, respectively. The putative venom genes of the two wasps showed no significant similarity or convergence. More venom genes were predicted in *D*. *collaris* VG than *C*. *vestalis* VG, especially hydrolase-coding genes. Differences in the types and quantities of putative venom genes shed light on different venom functions.

## Introduction

Hymenopteran parasitoids introduce venoms into their hosts at oviposition that facilitate development of their progeny. Venoms can cause paralysis, suppression of immune responses, modulation of the nutritional environment, and alteration of host development, either alone or in combination with other factors^1^. The venom components of Hymenopteran parasitoids are diverse, often consisting of a complex mixture of proteinaceous as well as nonproteinaceous biomolecules. Components can include neurotoxins, amines, small peptides, and mid- to high-molecular-weight enzymes^2^. The venom components of 17 parasitoid species, representing five families, have been analyzed. About 60 proteins found in parasitoid venoms share significant homology with proteins with known functions. However, no known functions exist for the vast majority of parasitoid venom proteins so their specific roles in parasitism are unknown^3^.

**Figure 1:**
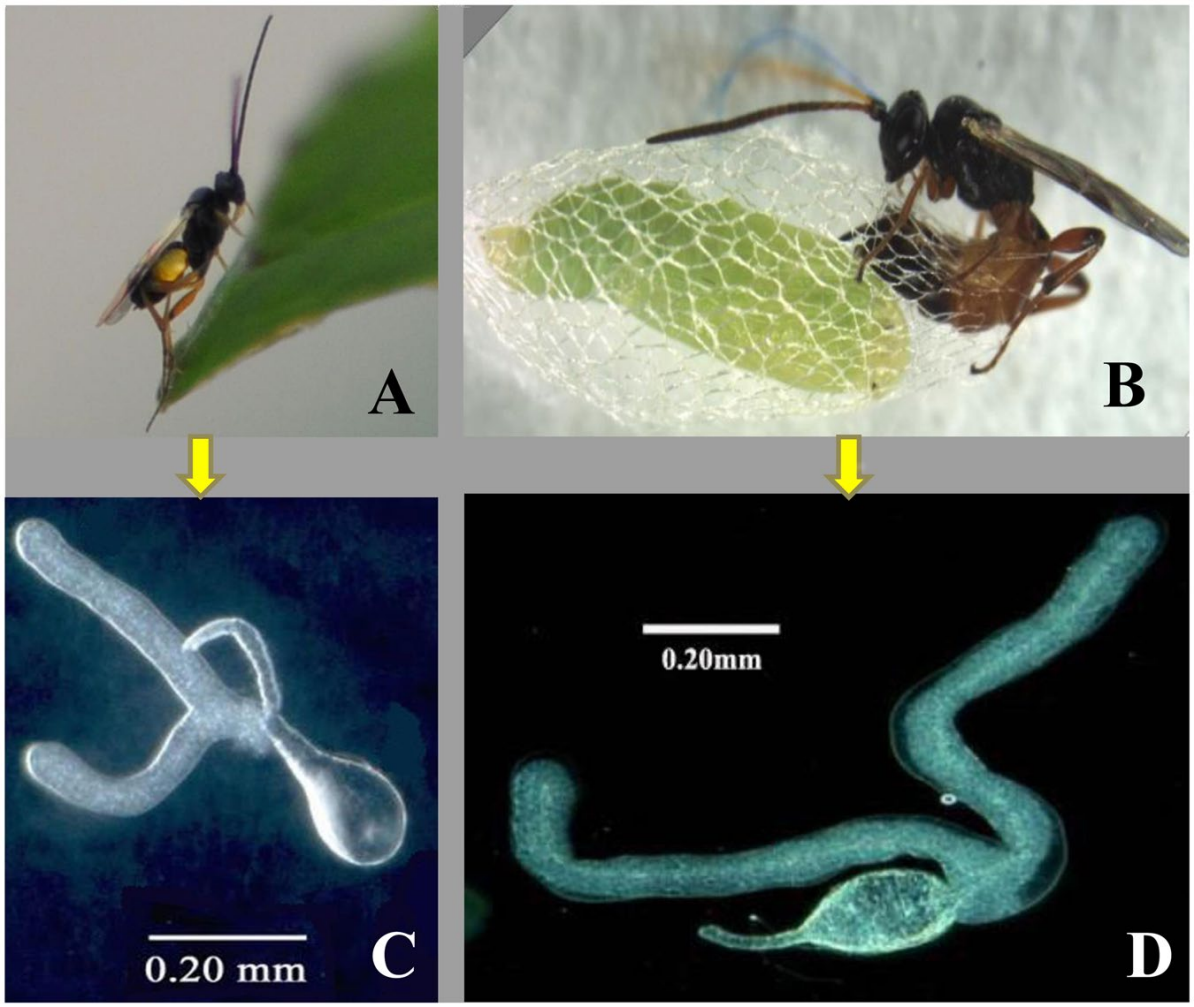
Two wasps and their venom apparatuses. (**A**) and (**C**) *C*. *vestalis* and its venom apparatus. (**B**) and (**D**) *D*. *collaris* and its venom apparatus.

*Cotesia vestalis* (Braconidae), a larval endoparasitoid, and *Diadromus collaris* (Ichneumonidae), a pupal endoparasitoid, have been recorded in many parts of the world as two of the most important biological control agents of the diamondback moth (DBM), *Plutella xylostella* (Plutellidae), the most significant cosmopolitan pest of crucifer vegetable crops (Fig. 1)^4, 5^. These two wasps both parasitize the DBM but use different arsenal combinations. *C*. *vestalis* possess all parasitic factors, such as venom, polydnavirus (PDV) and teratocytes originating from the serosal membrane that surrounds the developing embryo of the parasitoid, whereas *D*. *collaris* uses only venom for host parasitism^6–8^. Therefore, it seems that one parasitic weapon in *D*. *collaris* could complete the mission undertaken by three parasitic weapons in *C*. *vestalis*. Crude venom alone from *C*. *vestalis* has a limited effect on hemocytes and probably synergizes the effect of calyx fluid or polydnavirus^9^ while venom combined with other parasitic factors, such as PDVs can affect host protein metabolism, suppress immune responses, and cause parasitic castration by degenerating host testes^10, 11^. Venom of *D*. *collaris* can impair cell and humor-mediated immune responses of the host by changing the total number, morphology, and behavior of hemocytes and inhibiting the phenoloxidase activity of the hemolymph^12, 13^. Therefore, venom components and functions of the two wasps should be different and compatible with their specific parasitic lifestyles. Because the two wasps parasitize the same host, it was intriguing to compare their venom components which had not previously been studied.

Conventional methods of combining venom protein separation with bioactivity assays are time-consuming and low throughput while high throughput proteomics methods are dependent on genomic information^14^. It is difficult to collect adequate pure venom for proteome research^15^. The venom organ of parasitoid wasps is tiny (Fig. 1), the amount of venom is extremely limited, and contamination from the venom duct or venom glands (V﻿Gs) is inevitable when venom protein samples are prepared for proteome analysis. High-throughput transcriptomic analysis has recently been applied to study the VGs of parasitic wasps, and the feasibility of this technology has been proven^16–19^. Therefore, we also turned to VG transcriptome analysis for studying the venom components of the two wasp species.

In this study, we sequenced transcriptomes of VGs and bodies without venom glands (BWVGs) of these two species using Illumina technology. De novo assembly identified tens of thousands of distinct sequences. Genomic features of the two VGs were analyzed. Putative genes related to venom functions were discovered by secretory protein prediction and comparative transcriptome analysis. Our results provide insight into how venom functions in host-parasitoid interactions and will facilitate identification of more Hymenopteran venom genes.

## Results and Discussions

### Transcriptome overview

For VG and BWVG of *D*. *collaris*, Illumina sequencing yielded 26, 777, 782 and 88, 360, 364 reads with nucleotide sizes of 2,284, 178,940 and 7,139, 171,340 bp, respectively (Table 1). For VG and BWVG of *C*. *vestalis*, Illumina sequencing yielded 26, 234, 320 and 86,756,318 reads with nucleotide sizes of 2,199,844,980 and 7,203, 796,020 bp, respectively. All high-quality reads were assembled de novo by the Trinity program. We obtained 34,063 and 63,325 transcripts from VG and BWVG of *D*. *collaris* while 26, 066 and 51,641 transcripts from VG and BWVG of *C*. *vestalis*. After further process of sequence splicing and redundancy removal with sequence clustering software, we obtained 50,763 and 43,785 ALL-transcripts with an average length of 1005 nt and 828 nt for *D*. *collaris* and *C*. *vestalis*, respectively (Table 1). Next, we analyzed the length distribution of all-transcripts sequences. Although most sequences (>50%) were between 100 to 500 bp, 7186 sequences longer than 2,000 bp were identified in *D*. *collaris*. A similar trend was observed in *C*. *vestalis* (Supplementary Fig. S1A).

**Table 1:** Summary of the transcriptomes.

	DCBWVGs	DCVGs	DC	CVBWVGs	CVVGs	CV
Total number of reads	88,360,364	26,777,782	—	86,756,318	26,234,320	—
Total base pairs (bp)	7,139,171,340	2,284,178,940	—	7,203,796,020	2,199,844,980	—
GC percentage	47.53%	45.69%	—	41.93%	39%	—
Average read length (bp)	90	90	—	90	90	—
Total number of contigs	108,198	65,680	—	88,392	49,265	—
Mean length of contigs (bp)	409	305	—	372	284	—
Total unique sequences	63,325	34,063	50,763	51,641	26,066	43,785
Number of sequences in all-transcripts	28,394	48,725	—	41,796	32,775	—
Sequences with E-value <10^−5^	16,121	23,276	26,753	22,148	15,723	26,483

DCVGs: *D*. *collaris* venom glands; DCBWVGs: *D*. *collaris* bodies without venom glands; CVVGs: *C*. *vestalis* venom glands; CVBWVGs: *C*. *vestalis* bodies without venom glands.

**Figure 2:**
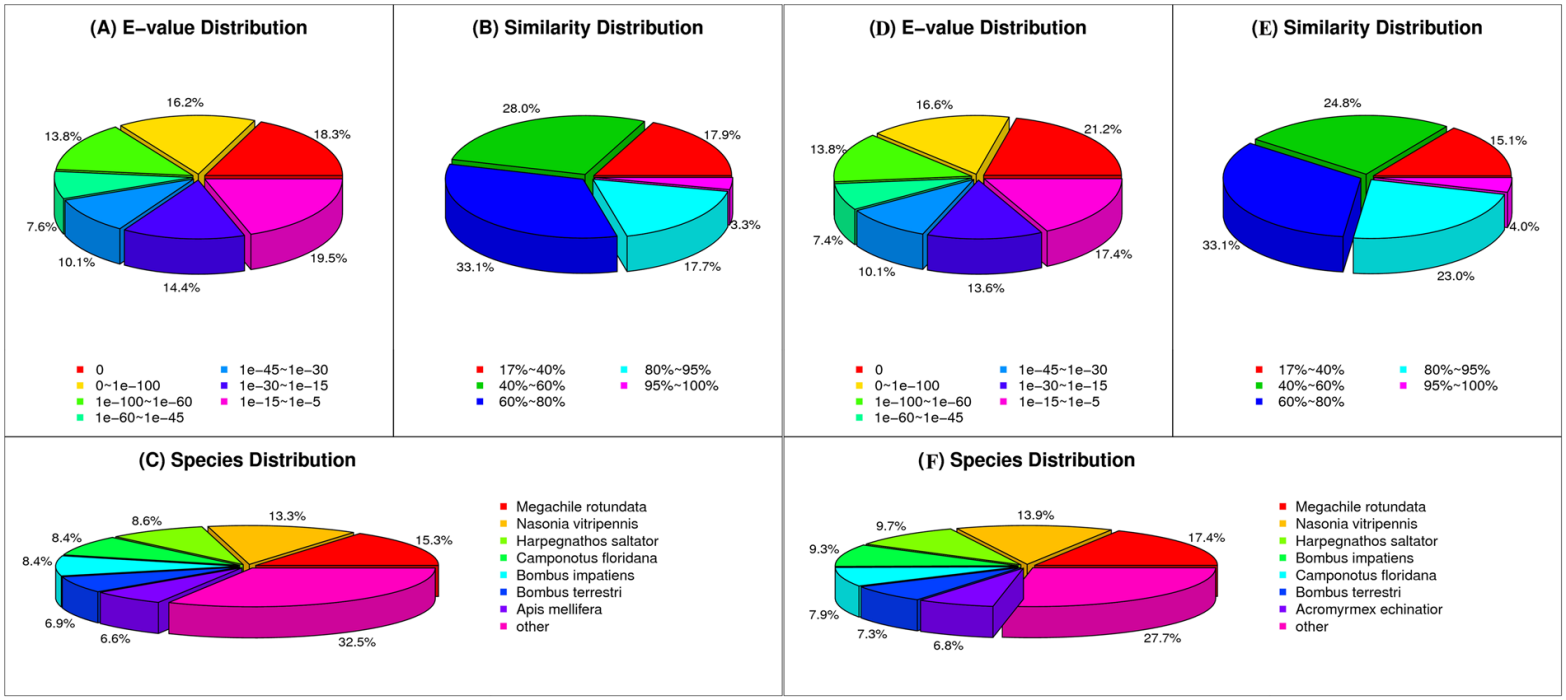
Characteristics of homology search of Illumina sequences against the nr database. (**A**) and (**D**): E-value distribution of BLAST hits for *D*. *collaris* all-transcripts and *C*. *vestalis* all-transcripts with a cut-off E-value of 1.0E^−5^. (**B**) and (**E**): Similarity distribution of the top BLAST hits for *D*. *collaris* all-transcripts and *C*. *vestalis* all-transcripts. (**C**) and (**F**): Species distribution is shown as a percentage of the total homologous sequences with an E-value of at least 1.0E^−5^ in *D*. *collaris* all- transcripts and *C*. *vestalis* all-transcripts. We used the first hit of each sequence for analysis.

For annotation, all-transcripts sequences were searched by BLASTx against the non-redundant (nr) NCBI database using a cut-off E-value of 10^−5^. 26,753 (53%) and 26,483 (60%) sequences returned an above cut-off BLAST result for *D*. *collaris* and *C*. *vestalis*. The proportion of sequences with matches in nr databases was greater among the longer assembled sequences (Supplementary Fig. S1B). The E-value distribution of best hits against the nr database showed that about 59% of the mapped sequences have strong homology (smaller than 1.0E^−50^), whereas 41% of the homolog sequences range from 1.0E^−5^ to 1.0E^−50^ in *D*. *collaris* (Fig. 2). All-transcripts sequences of *C*. *vestalis* has nearly the same E-value distribution pattern. Similarity distribution analysis shows that over 60% matches are more than 60% similar in *D*. *collaris* and *C*. *vestalis*. As to the species distribution, the two wasp transcriptomes were very similar. The highest percentage of unigenes of the two wasps matched the genes of the alfalfa leafcutter bee *Megachile rotundata*, followed by the jewel wasp *Nasonia vitripennis*, and the Jerdon’s jumping ant *Harpegnathos saltator* (Fig. 2).

### Gene Ontology (GO) and Kyoto Encyclopedia of Genes and Genomes (KEGG) analysis of venom gland genes

Among the all-transcripts, the transcripts having reads in VG or BWVG represented VG transcriptomes or BWVG transcriptomes, respectively. *D*. *collaris* and *C*. *vestalis* VG transcriptomes consisted of 32775 and 28394 transcripts, respectively (Table 1).

**Figure 3:**
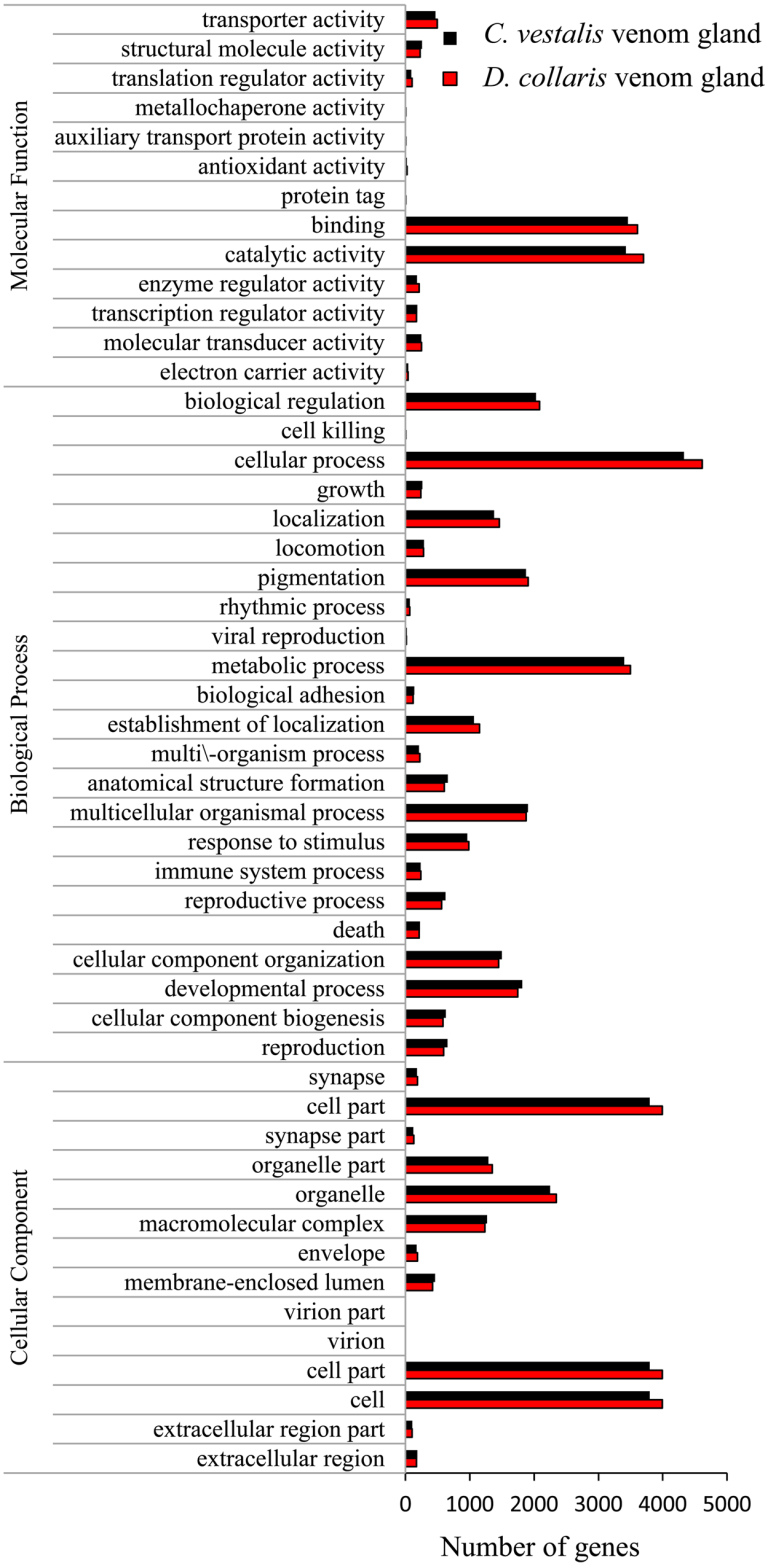
GO term distribution of venom gland genes at level two.

The GO classification system allows descriptions of gene products in terms of their associated biological processes, cellular components, and molecular functions. Overall, 7698 genes of *D*. *collaris* VG and 7189 genes of *C*. *vestalis* VG were assigned to GO terms. In both VGs, sequences to which GO categories were assigned had the greatest representation in ‘Cellular process’. For both VGs, in the three main divisions (cellular component, molecular function, and biological process) of the GO classification, the categories ‘Cell’, ‘Binding’, and ‘Cellular process’ were dominant, respectively (Fig. 3).

The distribution pattern of GO terms in the two VGs showed great similarity at levels 2, 3, 4, and 5 across GO categories with respect to the number of transcripts linked to each GO term. Pearson’s correlation coefficients were all significant (>0.99 with P-value ≪ 0.01 at three levels) either for the whole GO terms or each main division (Supplementary Table S1), indicating that the two VG transcriptomes had significantly similar function profiles or patterns. The semantic similarity of two GO term sets measured by G-SESAME was 0.75056 (Maximum value = 1), indicating relatively higher similarity.

To study the physiological characters of the VGs, statistically enriched GO terms were identified and analyzed compared to the whole-body transcriptome background. In the category of ‘Molecular Function’, ‘Catalytic activity’ was enriched in the two VGs at level two while ‘Binding’ was only enriched in *D*. *collaris* VG. Analysis of ‘Cell Component’ category enrichment indicated that ‘Organelle’, ‘Cell’, ‘Organelle part’, ‘Membrane-enclosed lumen’, and ‘Macromolecular complex’ were significantly enriched at level two in both VGs. For the category of ‘Biological Process’, ‘Metabolic process’ was enriched at level two in both VGs while ‘Cellular process’ was only enriched in *C*. *vestalis* VG at level two in the ‘Biological Process’ category (Supplementary Table S2).

The KEGG orthology (KO) is a classification system that provides an alternative functional annotation of genes by their associated biological pathways. A total of 7604 *D*. *collaris* and 7338 *C*. *vestalis* VG genes were assigned to KOs based on sequence homologies. *D*. *collaris* and *C*. *vestalis* VG genes were mapped to 252 and 256 KEGG pathways, respectively (Supplementary Table S3). A total of 252 pathways were shared by the two transcriptomes. Pathways ‘Asthma’, ‘Retinol metabolism’, ‘Porphyrin and chlorophyll metabolism’, and ‘Terpenoid backbone biosynthesis’ were only found in *D*. *collaris* VG. The pathways with the most representation in both VGs were ‘Metabolic pathways’, ‘spliceosome’, and ‘RNA transport’. In almost all pathways, the two transcriptomes had similar representations in the number of distinct annotations within each pathway. For the 252 shared pathways, Pearson’s correlation coefficient in percentages of transcript representations, indicated significant similarity in percentages of transcript representations (*r* > 0.97, p < 3.82 E-177) (Supplementary Table S3).

Enrichment analysis was also performed to identify the over-expressed pathways with the whole-body transcript distribution as background. Totally, 44 and 36 enriched pathways (*P* ≤ 5.0E^−3^) were identified in *D*. *collaris* VG and *C*. *vestalis* VG (Supplementary Table S4). Among them, 24 pathways were enriched in both VGs. At level four, ‘Spliceosome’, ‘Ubiquitin mediated proteolysis’, ‘Tuberculosis’, and ‘RNA degradation’ were the top four enriched pathways in *D*. *collaris* VG. ‘Spliceosome’, ‘Protein processing in endoplasmic reticulum’, and ‘Proteasome’, were the top 3 enriched pathways in *C*. *vestalis* VG. At level two, ‘Genetic information processing’ and ‘Metabolism’ were dominant in both VGs (Supplementary Table S4).

VGs are specific organs for production of venom macromolecules and secretions, and these organs have a high level of metabolic activity. Previous research demonstrated that the ultrastructure of the secretory units of the gland tubules in *D*. *collaris* was consistent with the model of a type III gland cell, which was quite similar to the *C*. *vestalis* VG and VGs described in other parasitoids^20–24^. The apparatuses of the two VG cells were abundant and included Golgi apparatus, rough endoplasmic reticulum, and mitochondria. The abundance of these components was consistent with intense protein synthesis and suggests vigorous activities in the VG cells^6, 20^. Indeed, many over-expressed GO terms and KEGG pathways reflected these features. Most enriched GO terms in ‘Cell Component’ such as ‘Organelle’, ‘Organelle part’, and ‘Membrane-enclosed lumen’ were consistent with the observation of abundant apparatuses in gland cells. In the meantime, the enrichment of the pathways of ‘Genetic information processing’ and ‘Metabolism’ and the over-expressed GO terms of ‘Metabolic process’ and ‘Macromolecular complex’ were also consistent with the active processes of macromolecule biosynthesis and catabolism in gland cells. Interestingly, ‘Catalytic activity’ and ‘Binding’ enriched in ‘Molecular Function’ were also the most represented functional categories assigned to the VG ESTs from the saw-scaled viper, *Echis ocellatus* ^25^, the solitary hunting wasp species, *Orancistrocerus drewseni* ^26^, the endoparasitic wasp, *Chelonus inanitus* ^27^, and the ant, *Tetramorium bicarinatum* ^19^. Vincent *et al*.^27^ suggested that catalytic activity and binding categories thus may constitute a hallmark of the VG transcriptomes analyzed to date^27^.

In conclusion, the obvious similarity in distribution profiles and enrichment results of GO terms and KEGG pathways between the two wasp VGs might reflect the similar secretory structure and function of two VGs at the genetic level. These data will help draw a general pattern for the biosynthesis and secretion of venom proteins of two VGs.

### Differently expressed genes in VG compared to BWVG

**Figure 4:**
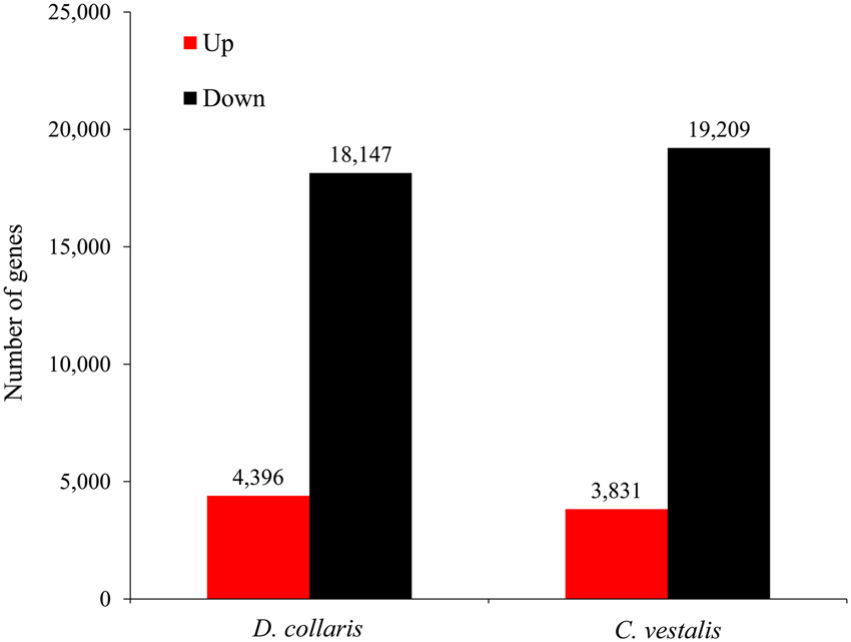
Changes in gene expression profiles between venom glands (VGs) and bodies without venom glands (BWVGs).

Differences in reads frequencies in the VG and BWVG libraries were used to estimate differences in gene expression level between two libraries. We identified 22,543 and 23,040 genes that were expressed at significantly different levels between VG and BWVG in *D*. *collaris* and *C*. *vestalis* (Fig. 4 and Supplementary Table S5). Of these, 4396 were up-regulated and 18147 were down-regulated in the *D*. *collaris* VG while 3831 genes were up-regulated and 19,209 genes were down-regulated in the *C*. *vestalis* VG (Fig. 4 and Supplementary Table S5).

We also identified 1,595 and 1,461 VG specific genes in up-regulated genes that were only expressed in *D*. *collaris* and *C*. *vestalis* VGs. Of these, 422 *D*. *collaris* (26%) and 581 *C*. *vestalis* (40%) genes could be annotated based on alignments to the nr database (Supplementary Tables S6 and S7). Many were highly expressed. FPKMs (Fragments Per kb per Million fragments) of 38 *D*. *collaris* VG and 6 *C*. *vestalis* VG specific genes were >1000. Only 19 *D*. *collaris* VG and 1 *C*. *vestalis* VG highly expressed genes were annotated while the rest showed no similarity to any known proteins. CL3939. Contig3_All of *D*. *collaris* VG that matched a protein inhibitor and Unigene30513_All of *C*. *vestalis* that was similar to a venom protein were the two most highly expressed annotated genes. CL2424. Contig1_All of *D*. *collaris* VG with a 91787.8632 FPKM and Unigene32174_All of *C*. *vestalis* VG with a 62339.5501 FPKM, with no annotation and the most highly expressed, should be completely new proteins. Interestingly, the annotation rate was obviously lower in gland specific genes than other VG genes. Perhaps these genes, probably the most possible VG function related, evolved and diverged more rapidly. Next, these VG specific genes were classified through GO and KEGG annotation. At level 2, ‘Catalytic activity’ and ‘Binding’ were dominant in ‘Molecular Function’, providing the same result as in the two VG transcriptomes mentioned above. KEGG annotations of VG specific genes of the two wasps differed considerably at level 3 (Supplementary Tables S6 and S7). ‘Metabolic pathways’ contained the most VG specific genes of *D*. *collaris* VG while ‘Ribosome’ was the highest in occurrence in the VG specific genes of the *C*. *vestalis* VG. This is consistent with the function of active protein synthesis and metabolic activities in the VG.

To validate the gene expression data obtained through statistical comparison of FPKM value, we compared the gene expression profiles of VG and BWVG using quantitative PCR (qPCR). A good correlation was obtained for the results of RNA-Seq and qPCR analysis in 10 genes from each wasp (Supplementary Tables S8 and S9).

### Secreted protein prediction and function analysis

Venoms were secreted by VG cells from the parasitoid wasps. Therefore, venom proteins are expected with signal peptides in their amino acid sequences. A total of 532 and 457 potential secretory proteins were identified in the *D*. *collaris* VG and the *C*. *vestalis* VG while 499 and 419 had annotations in the nr database (Supplementary Table S10). These genes, encoding potential secretory proteins, were defined as putative venom genes of the two wasps and further analyzed.

A total of 116 *D*. *collaris* VG and 70 *C*. *vestalis* VG secretory proteins were homologs of known Hymenopteran venom proteins. However, most putative secretory proteins showed no significant similarity to known venom proteins. There exists a great possibility that these secretory proteins represent novel venom proteins for each wasp. Among all VG genes, significantly more homologous sequences of known Hymenopteran venom proteins were in the putative secreted proteins (21.8% in *D*. *collaris* VG and 15.3% in *C*. *vestalis* VG) than those in non-secretory sequences (3.5% in *D*. *collaris* VG and 1.42% in *C*. *vestalis* VG), especially in the up-regulated subgroups of secretory proteins (27% in *D*. *collaris* VG and 21.4% in *C*. *vestalis* VG) (Supplementary Table S10).

Immunological similarities exist across venoms of many Hymenopteran species. Antibodies raised against *Chelonus* nr. *curvimaculatus* (Braconidae) venom reacted with venom proteins from the Formicidae, Vespidae, and Apidae. Venom proteins from mostly primitive parasitic wasps and ants showed much higher cross-reactivity than aculeate wasp and bee venom^28^. Conversely, four venom proteins from the ectoparasitoid wasp *Eupelmus orientalis* were recognized by polyclonal antibodies raised against venom proteins from *Apis mellifera* ^1, 29^. Recently, antibodies against *P*. *puparum* calreticulin, GOBP-like venom protein, venom protein U, serine protease 22, and serine protease homolog 29 were found to cross detect the venom proteins in *N*. *vitripennis* ^16^. Comparative analysis indicates that venoms of social Hymenoptera species are qualitatively similar with venoms produced by parasitic aculeates^30–32^. Many types of venom proteins in parasitic wasps are also present in social and solitary wasps or bees as allergens such as antigen 5 and acid phosphatase^33^. These immunological similarities, due to similar amino acid identity or similar post-translational modifications, suggest evolutionary conservation of venom composition and perhaps functionality in spite of their apparently different functions^34^. Therefore, the continuity of venom protein evolution could be the reason for this great similarity with other Hymenopteran venom proteins in putative secreted proteins and provides further evidence that our approach has led to the successful identification of venom proteins.

*D*. *collaris* VG has 116 putative secretory proteins sharing homology with venom components detected in other wasps, including pupal endoparasitoids (Supplementary Table S10). Pupal endoparasitoids like *D*. *collaris* are restricted primarily to a few subfamilies of the Ichneumonidae^30, 35, 36^. The best-studied species is *Pimpla hypochondriaca*, a solitary pupal endoparasitoid, which injects a venom that paralyzes and immunosuppresses its Lepidopteran host^30, 37^. Neither embryos nor feeding-stage larvae of *P*. *hypochondriaca* appear to play a role in altering host development or immune defenses^30^. A number of venom genes have been identified by random sequencing of the venom gland cDNA library and classical bottom-up proteomic approaches^2, 37, 38^. *D*. *collaris* as a solitary pupal endoparasitoid lives a parasitic life similar to *P*. *hypochondriaca* and thus its progeny face the similar challenges. Because there are no additional parasitoid-associated factors, such as PDVs and teratocytes, in *D*. *collaris*, venom may play a role similar to that of *P*. *hypochondriaca* venom in host immune suppression and host regulation^12, 13^. A similarity search of putative secreted proteins against *P*. *hypochondriaca* venom proteins returned matches to trehalase, metalloprotease, cysteine-rich venom protein 6, cysteine-rich venom protein 2, and two other proteins. This indicates venom components with potentially similar functions in *D*. *collaris* venom.

*C*. *vestalis* VG has 70 putative secretory proteins significantly matched to venom genes of other wasp species, including PDV-producing parasitoids (Supplementary Table S10). For many PDV-producing parasitoids like *C*. *vestalis*, venom proteins are required for PDV function or to provide synergistic effects. This ranges from complete independence of some ichneumonid PDVs (ichnoviruses) to variable dependency of braconid PDVs (bracoviruses) on venom^2^. Yu *et al*.^9^ found that *C*. *vestalis* venom alone may be insufficient to suppress the host immune system, but it might synergize the effects of calyx fluid or polydnavirus as in other insect-host systems^9^. A number of putative secreted proteins in the *C*. *vestalis* VG transcriptome showed evident similarity to venom proteins from PDV-producing parasitoids such as *Cotesia rubecula*. For example, the Unigene32176_All coded for a protein most similar to “Venom protein Vn4.6” which seemed to interfere with the activation of host hemolymph prophenoloxidase^39^. Intriguingly, Unigene32176_All, with 32,734 FPKM, was also the most frequently sequenced transcript with annotation in either *C*. *vestalis* VG or genes coding for secretory proteins. CL1620. Contig8_All matched to venom protein Vn50 and Unigene8653_All had a 0 e-value against calreticulin in *C*. *rubecula* venom. This might indicate similar venom functions present in *C*. *vestalis* venom.

Many venom proteins have been discovered to suppress host immunity including humoral and cellular immunity, and dominant in quantity among all the venom proteins^3^. Host immune suppression by the two wasp venoms has been primarily studied as mentioned above in the introduction part. Putative venom proteins of the two parasitoids contain homologs of immune-suppressing proteins (Supplementary Table S10). Proteins similar to immune-suppressing proteins, including Vn50^40^, calreticulin^41^, and super oxide dismutase^42^, were found in putative venom proteins of two wasps. Proteins with significant similarity to Vn4.6^39^ and serpin^43^ also existed in *C*. *vestalis* potential secretory venom proteins. These proteins might help the parasitoids escape the host immunity responses and will be the focus of our study in the future. For the rest of putative venom-coding genes, either venom gene homologies or novel venom genes, their potential roles in interactions with the host should also be taken into consideration during the studies in the future.

All genes encoding secretory proteins consist of 111 up-regulated and 287 down-regulated genes in *D*. *collaris* VG, while 56 up-regulated and 312 down-regulated genes in *C*. *vestalis* VG (Supplementary Table S10). Previous studies of venom genes have demonstrated that most venom coding genes were either up-regulated or even venom tissue specific^39, 41–47^. Therefore, these secreted proteins encoded by up-regulated transcripts are likely to be real venom proteins. Among the up - regulated genes, 34 and 11 genes were only expressed in *D*. *collaris* VG and *C*. *vestalis* VG including some highly-expressed transcripts (FPKM > 1000). These included CL2038. Contig3_All, Unigene35108_All, and CL3939. Contig1_All in *D*. *collaris* VG and Unigene32174_All and Unigene30513_All in *C*. *vestalis* VG. Although several VG specific genes still have no function annotations, these genes should be the most probable venom genes (Supplementary Table S10).

**Figure 5:**
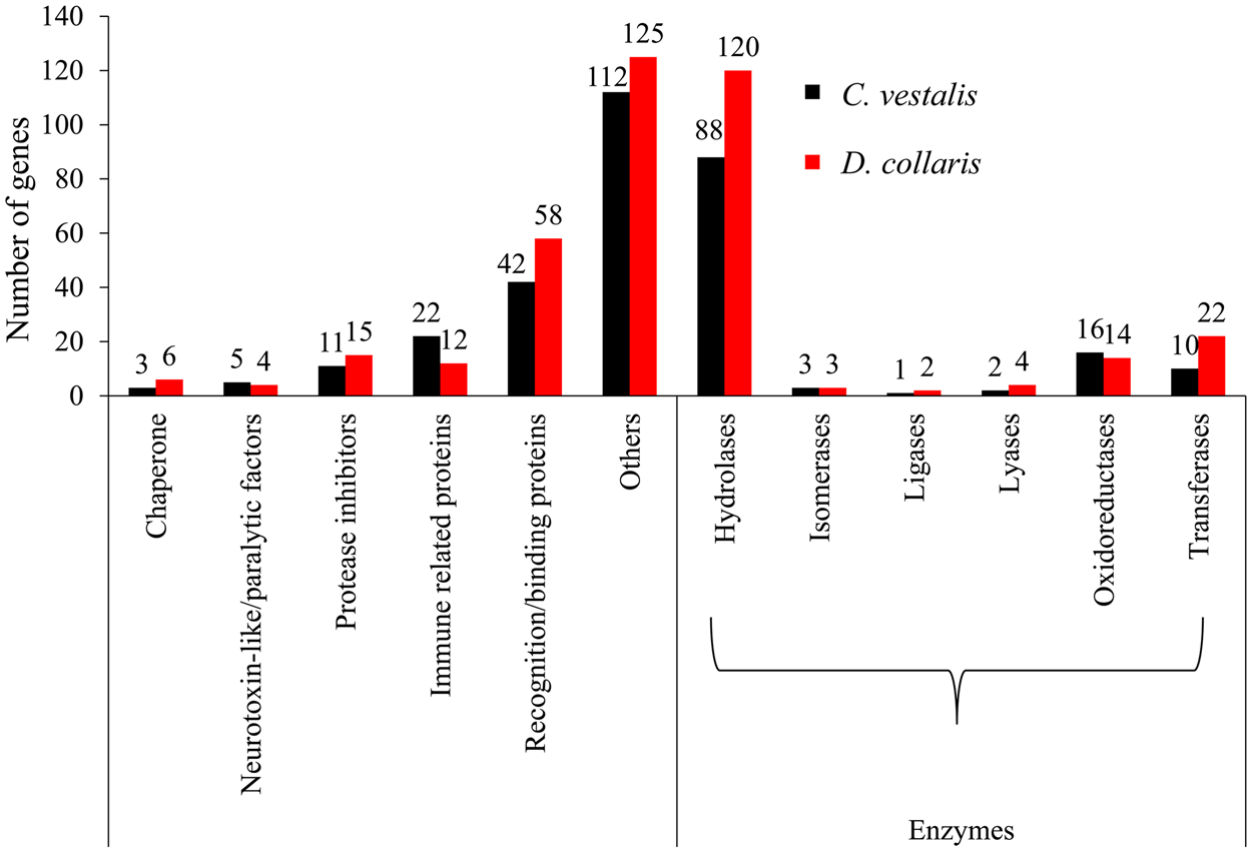
Function classification of putative secretory proteins.

A total of 182 and 177 distinct domains were identified in the putative secreted proteins of *D*. *collaris* VG and *C*. *vestalis* VG, respectively (Supplementary Table S10). Of these, 94 domains were shared. All of these putative secretory proteins of two wasp species appeared to fall into seven different broad functional categories by combining domain and nr annotation data according to Poirie *et al*.^3^. These categories included (1) enzymes; (2) protease inhibitors; (3) immune related proteins; (4) recognition/binding proteins; (5) neurotoxin-like/paralytic factors; (6) chaperones, and (7) others (Fig. 5). Apparently more secretory proteins were classified into the enzyme category in *D*. *collaris* VG (170) than *C*. *vestalis* VG (125). The enzyme category was further classified into subcategories and compared based on their specific functions (Supplementary Table S10). Significantly more hydrolases were in *D*. *collaris* VG secretory proteins (120) than *C*. *vestalis* VG secretory proteins (88), such as peptidase (17 in *D*. *collaris* VG and six in *C*. *vestalis* VG), esterase (16 in *D*. *collaris* VG and six in *C*. *vestalis* VG) and trehalase (four in *D*. *collaris* VG and 0 in *C*. *vestalis* VG) (Fig. 5 and Supplementary Table S10).

**Figure 6:**
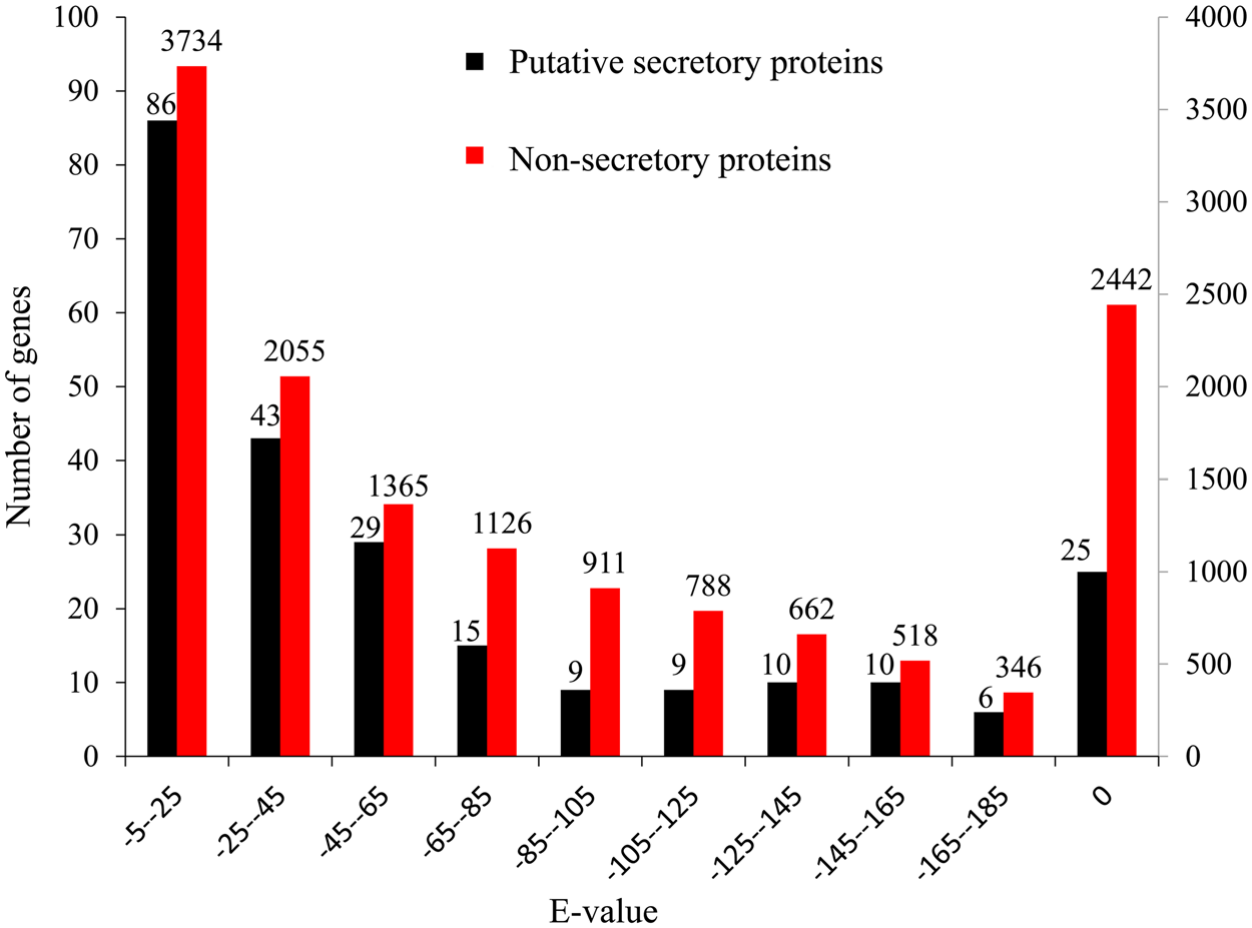
Sequence similarities of secreted proteins and non-secreted proteins between two venom glands.

Sequence similarities of secreted proteins and non-secreted body proteins between the two VGs were compared using blastp^48^. Results showed that the e-value distribution between secreted proteins of the two VGs resembled that between non-secreted proteins (Pearson coefficient = 0.93, p < 0.01) (Fig. 6).

Most putative venom genes were different between two wasp species and no significant similarity was discovered between putative venom genes of the two wasps (Supplementary Table S10). Convergent evolution apparently did not happen in the two venoms even though both venoms are used to suppress immune reactions of the same host^9, 12^. The parasitization of different development phases by two wasps, wasp phylogeny, and how the venom interacts with host physiology might have had greater influence on the venom components. Recent studies on the wasp *Leptopilina* species (Figitidae) indicated that venom composition could mainly differ even between closely-related species parasitizing the same host^49, 50^.

More putative venom genes, especially genes coding for hydrolases, were predicted in the *D*. *collaris* VG transcriptome than in the *C*. *vestalis* VG transcriptome. Many factors such as the limited number of known venom genes or incomplete N-terminal unigene regions could have affected the prediction results. However, considering that *C*. *vestalis* possesses three parasitic factors while *D*. *collaris* has only venom, venom from *D*. *collaris* ought to have more functions to accomplish successful parasitism. Li *et al*.^12^ reported that venom of *D*. *collaris* might be able to destroy the structure of the host fat body and adipocytes to release nutrition for progeny development. Hydrolases should play a role in this process^12^. However, no proteins with any similar function were discovered in *C*. *vestalis* venom. Therefore, the advantage of *D*. *collaris* VG in putative venom genes, especially hydrolase related genes, may not be a mere coincidence.

## Conclusion

We sequenced the transcriptomes of two endoparasitoids of *P*. *xylostella* using Illumina sequencing technology. A great number of unique transcripts were assembled and annotated. An evident similarity between the two VGs was discovered in the distribution profiles of GO terms and KEGG pathways in the two VG transcriptomes. Enriched GO terms and pathways of the two VGs and VG specific genes were consistent with active activities of protein biosynthesis in the VGs. Putative venom genes of the two wasps showed no obvious similarity or convergence although the wasps parasitize different stages of the same host. More venom genes were predicted in *D*. *collaris* VG than *C*. *vestalis* VG, especially hydrolase-coding genes. The differences in the types and quantities of putative venom genes between the two wasp species shed some light on divergent venom functions of the two endoparasitoids. We speculated that the divergence of two venom gland transcriptomes might suggest that the evolution of two venom glands has led to the diversity of venom components and functions adapting to specific parasitic lifestyles while their similarities in distribution profiles and enrichment results of GO terms and KEGG pathways may reflect their origin from the common ancestor and retain a potential conserved transcriptome profile for venom production. Taken together, our results provide an invaluable resource for the identification of additional Hymenopteran venom genes and will contribute to the understanding of how venom functions in host-parasitoid interactions.

## Methods

### Insect rearing and sample preparation

Parasitoids and the host *P*. *xylostella* were maintained as previously described^5, 6^. Briefly, an abundance of hosts at proper stages were exposed to each parasitoid for parasitization. Larvae parasitized by *C*. *vestalis* were reared on cabbage while pupae parasitized by *D*. *collaris* were maintained in a container until emergence of adult wasps. Adult wasps were fed 20% honey water. Both parasitoid species and their host were maintained at 25 ± 1 °C, 65% relative humidity under a 14 h light:10 h dark photoperiod. VGs were dissected from 0–7 day old mated female wasps, and then both VGs and BWVGs were collected in 1.5-ml microtubes containing Trizol reagent (Invitrogen Life technologies, CA, USA), respectively. After homogenization, samples were stored in a −70 °C refrigerator. The total RNA was extracted using Trizol reagent according to the manufacture’s protocol.

### RNA isolation and library preparation

Total RNA was extracted from VG and BWVG using TRIZOL reagent according to the manufacturer’s protocol. RNA integrity was confirmed using the 2100 Bioanalyzer (Agilent Technologies) with a minimum RNA integrated number value of 8. The samples for transcriptome analysis were prepared using Illumina’s kit following manufacturer’s instructions. Briefly, poly(A) mRNA was purified from 20 μg of total RNA using oligo(dT) magnetic beads and fragmented into short sequence by fragmentation buffer. The cleaved poly(A) RNA fragments were used for first strand cDNA synthesis using random hexamer-primer followed by second strand cDNA synthesis using RNaseH and DNA polymerase I. After the end repair and ligation of adaptors, the products were purified and enriched with PCR to create a cDNA library.

### Transcriptome assembly and annotation

Four cDNA libraries were sequenced at the Beijing Genome Institute (Shenzhen, China) on the Illumina HiSeq™ 2000 platform. Transcriptome de novo assembly was accomplished with assembling program - Trinity^51^. After removal of adaptor sequences, empty reads and low quality sequences, sequences from the two libraries were assembled into contigs. Then the reads were mapped back to contigs. Contigs from the same transcript were detected and further assembled with paired-end reads. For transcripts from each of the two libraries, TGIC^52^ and Phrap^53^ were used to assemble to non-redundant (nr) all-transcripts by gene clustering.

Transcript sequences were first aligned by blastx (http://blast.ncbi.nlm.nih.gov/Blast.cgi) against protein databases like nr, Swiss-Prot, and KEGG, retrieving proteins with the highest similarity with the given transcripts along with their protein functional annotations. With nr annotation, GO annotation, and functional classification for transcripts were analyzed using Blast2GO^54, 55^ and WEGO software^56^. Orientation and coding sequence (CDS) of sequences which had no hits in blast were predicted using ESTScan^57^. Transcript expression level was calculated using the FPKM method (Fragments Per kb per Million reads).

Pearson’s correlation coefficient was used to evaluate the correlation values with respect to the percentages of transcript representations linked to each GO term or KEGG biological pathway between two transcriptomes.

G-SESAME (http://bioinformatics.clemson.edu/G-SESAME/index.php) was used to measure the semantic similarity of GO term sets^58, 59^.

### Secretory protein prediction and protein domain identification

The BLAST results were used to extract CDSs from transcripts. The CDS of the transcripts that has no significant hit in BLAST search were predicted by ESTScan^57^. Prediction of signal peptides was carried out using the SignalP 3.0 Server (http://www.cbs.dtu.dk/services/SignalP-3.0/)^60^. To remove sequences that also contained a transmembrane domain in addition to the signal peptide, we used TMHMM Server (http://www.cbs.dtu.dk/services/TMHMM/) to predict transmembrane region. The putative protein that has a signal peptide and with no or one transmembrane domain would be considered as a potential secreted protein^61, 62^.

Protein domains were identified by searching the Pfam database (http://pfam.xfam.org) using the HMMER web server (http://www.ebi.ac.uk/Tools/hmmer/)^63, 64^.

### Identification of statistically enriched ontologies and pathways

The hypergeometric test was used to measure significantly enriched GO terms in the target gene groups in comparison with the background^62, 65^. The calculating formula used was $$p=1-{\sum }_{i=0}^{m-1}\frac{(\begin{matrix}M\\ i\end{matrix})(\begin{matrix}N-M\\ n-i\end{matrix})}{(\begin{matrix}N\\ n\end{matrix})}$$, where N is the number of all genes with GO annotation; n is the number of differentially expressed genes (DEGs) in N; M is the number of genes that are annotated to a certain GO terms; and m is the number of DEGs in M. The GO terms with the p-value cut-off of 5.0E^−3^ were deemed to be enriched. In addition, to identify the enriched pathways, the hypergeometric test was used similarly to measure the relative coverage of the annotated KEGG orthologous groups of a pathway in the background, and pathways with a p-value cut-off of 5.0E^−3^ were considered as enriched^66^.

In this paper, the wasp transcriptome means the all-transcripts. And the transcripts having reads in VG or BWVG represented VG transcriptome or BWVG transcriptome, respectively.

### Identification of differentially expressed genes

The expression differences between two samples were calculated with the FDR (false discovery rate) method. The FDR was applied to determine the threshold of the P-value in multiple tests and analyses^67^. An FDR < 0.001 and an absolute value of log2Ratio ≥ 1 were used as the threshold to judge the significance of gene expression differences.

### Quantitative real-time PCR (qRT-PCR) analysis

To confirm the results of the FPKM comparison, the expression profiles of 10 selected genes were measured using qPCR. Total RNAs of VG and BWVG were extracted using the SV Total RNA Isolation System (Promega, Fitchburg, USA). One microgram of RNAwas reverse transcribed for first-strand cDNA synthesis with the ReverTra Ace qPCR RT Kit (TOYOBO, Osaka, Japan). qRT-PCR was performed in ABI7500 Real-Time System (Applied Biosystems, Foster City, CA, USA) using SYBR Premix Ex Taq TM II (Takara, Shiga, Japan). The cycling parameters were 95 °C for 60 seconds followed by 40 cycles of 95 °C for 15 s and 60 °C for 35 s. For each gene, three biological replicates were analyzed and the average threshold cycle (C_*t*_) was calculated. The results were normalized to the expression level of the *C*. *vestalis* 18S rRNA gene (GenBank accession number: JX399880) and *D*. *collaris* 18S rRNA gene (GenBank accession number: KX912696). Finally, the relative expression level was calculated using the 2^−△△Ct^ method^68^.

### Data deposition

The four data sets of Illumina sequencing are available at the NCBI Short Read Archive (SRA) with the accession number: SRR1022346 (*D*. *collaris* VG), SRR4294717 (*D*. *collaris* BWVG), SRR1032213 (*C*. *vestalis* VG) and SRR3948414 (*C*. *vestalis* BWVG). The assembled sequences have been deposited in the NCBI’s TSA database: GEZZ00000000 (*D*. *collaris* all-transcripts) and GFAF00000000 (*C*. *vestalis* all-transcripts).

## Electronic supplementary material

Suppl Figure S1

Suppl Table S1

Suppl Table S2

Suppl Table S3

Suppl Table S4

Suppl Table S5

Suppl Table S6

Suppl Table S7

Suppl Table S8

Suppl Table S9

Suppl Table S10
