# Supplementary material for: Comparative transcriptome analysis of venom glands from Cotesia vestalis and Diadromus collaris, two endoparasitoids of the host Plutella xylostella
Source: Sci Rep. 2017 May 2;7:1298. doi: 10.1038/s41598-017-01383-2 (PMC5431001; doi:10.1038/s41598-017-01383-2)
Supplement: Supplementary file 1 — Suppl Figure S1 [file 41598_2017_1383_MOESM1_ESM.pdf]

Comparative transcriptome analysis of venom glands from *Cotesia vestalis* and *Diadromus collaris*, two endoparasitoids of the host *Plutella xylostella*

Wei Zhao [a,b], Min Shi [a,b], Xi-qian Ye [a,b], Fei Li [a,b], Xiao-wei Wang [a,b], Xue-xin Chen [a,b,c,*]

[a] Institute of Insect Sciences, Zhejiang University, 866 Yuhangtang Road, Hangzhou 310058, China

[b] Ministry of Agriculture Key Lab of Agricultural Entomology, Zhejiang University, 866 Yuhangtang Road, Hangzhou 310058, China

[c] State Key Lab of Rice Biology, Zhejiang University, 866 Yuhangtang Road, Hangzhou 310058, China

***Corresponding author:** *xxchen@zju.edu.cn, phone number: +86 571 88982868*

A

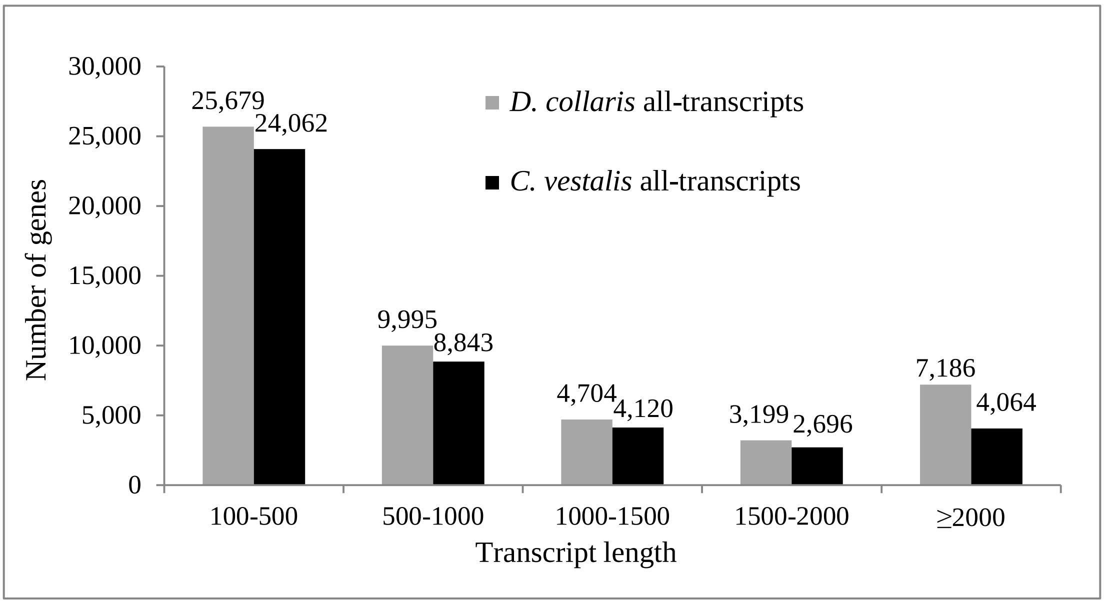

B

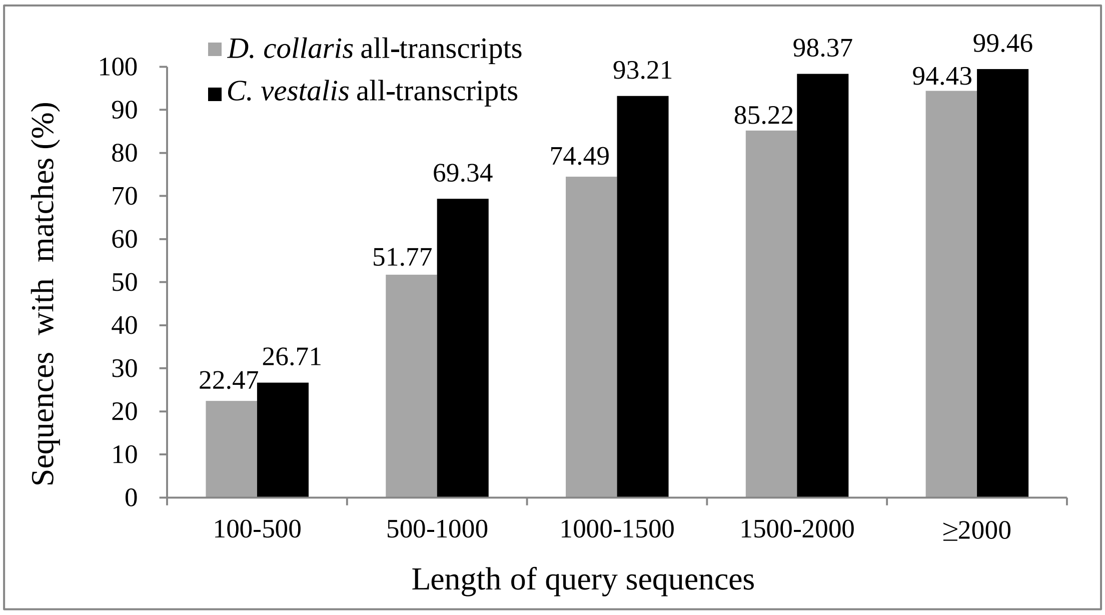

Figure S1. Size distribution for all-transcripts (A) and effect of query sequence length on the percentage of sequences for which significant matches were found (B).
